# Transependymal Cerebrospinal Fluid Flow: Opportunity for Drug Delivery?

**DOI:** 10.1007/s12035-017-0501-y

**Published:** 2017-04-28

**Authors:** João Casaca-Carreira, Yasin Temel, Sarah-Anna Hescham, Ali Jahanshahi

**Affiliations:** 1 School for Mental Health and Neuroscience, Maastricht University, Maastricht, the Netherlands; 2 Department of Neurosurgery, Maastricht University Medical Center, Maastricht, the Netherlands; 3 European Graduate School of Neuroscience (EURON), Maastricht, the Netherlands

**Keywords:** Cerebrospinal fluid, Intracerebroventricular drug delivery, Ependyma, Transependymal CSF flow

## Abstract

Drug delivery to the central nervous system (CNS) is complicated by the blood-brain barrier. As a result, many agents that are found to be potentially effective at their site of action cannot be sufficiently or effectively delivered to the CNS and therefore have been discarded and not developed further for clinical use, leaving many CNS diseases untreated. One way to overcome this obstacle is intracerebroventricular (ICV) delivery of the therapeutics directly to cerebrospinal fluid (CSF). Recent experimental and clinical findings reveal that CSF flows from the ventricles throughout the parenchyma towards the subarachnoid space also named minor CSF pathway, while earlier, it was suggested that only in pathological conditions such as hydrocephalus this form of CSF flow occurs. This transependymal flow of CSF provides a route to distribute ICV-infused drugs throughout the brain. More insight on transependymal CSF flow will direct more rational to ICV drug delivery and broaden its clinical indications in managing CNS diseases.

## Introduction

Central nervous system (CNS) disorders are currently estimated to affect hundreds of millions of people worldwide and represent a major public health problem [1]. Pharmacotherapy is often the gold-standard approach for a vast number of CNS disorders. However, systemic pharmacotherapy is a daunting challenge due to the presence of unique protective barriers, the blood-brain barrier (BBB) and blood-cerebrospinal fluid (CSF) barrier of the CNS [2]. In theory, many CNS drugs should be effective at their site of action. However, these agents fail to cross the BBB following systemic administration or very high drug doses are needed to achieve the desired efficacy [3]. The latter can lead to side effects or even toxicity. The necessity of delivering therapeutic agents into the CNS without augmenting the systemic concentration has resulted in developing new methods of CNS drug delivery. So far, three main ways have been developed: invasive, noninvasive, and miscellaneous approaches [3]. Here, we will elaborate upon the invasive methods.

Local drug delivery to the brain tissue or the ventricular system has been extensively used [4–7]. The ventricular system is filled with CSF, which can either hinder or facilitate drug delivery. The high turnover of the CSF that is meant to clear away metabolites can also rapidly clear drugs from the CNS and thereby reduce treatment efficacy. Conversely, some of the natural features of this circulating fluid have been used as advantages for the delivery of newly developed therapeutics into the CNS. CSF dynamics has been a topic of active research over the last century, in basic research as well as in clinical studies [8]. The actual perspective on CSF dynamics is different, and sometimes, evidence seems to be contradictory and paradoxical. Here, we aim to characterize and elaborate upon a relatively neglected form of CSF flow based on experimental and clinical data, which might change the concept of drug delivery in future applications. The present review will therefore first describe the factual knowledge about the physiology and circulation of the CSF. Subsequently, current insights on transependymal CSF flow are presented and discussed. Thereafter, original pre-clinical and experimental studies providing supporting data on this process are discussed. Finally, the possible clinical applications of this approach are outlined.

## The Physiology of CSF

Defined as a clear and bright fluid, CSF fills the ventricular system and subarachnoid space and has protective function since it serves as a hydraulic cushion for the brain and spinal cord [9]. Being the main circulating fluid in the CNS, it has specific metabolic, nutritional, immunologic, and scavenging functions [9]. CSF originates from cerebral arterial blood by active filtration and circulates along natural fluid passages and pools before being eventually absorbed into brain venous blood [10]. Among several important roles, the undisturbed circulation of CSF is essential for homeostasis in the CNS. CSF also acts as cushion for the CNS, because according to Archimedes’ principle, the brain and spinal cord lose their relative weight by floating in the CSF. Consequently, in a mechanical impact, the brain and spinal cord are more protected from injury. Moreover, the free circulation of CSF neutralizes the intracranial pressure gradients and eliminates the risk of volume shifts or herniation [10].

In humans, the normal CSF pressure is approximately 3 to 5 mmHg higher than the pressure in the venous sinuses, which creates a driving force for the outflow of CSF from the subarachnoid space across the basal membrane of the cells lining the arachnoid granulations and eventually across the apical membrane into the venous sinuses [11, 12]. Several physiological and pathological factors influence CSF pressure, which can vary with age [13]. The CSF drains via perineural space to the lymphatic system, via transependymal–interstitial to the perivascular subpial space, and via epithelium of the choroid plexus to the fenestrated capillaries and finally to the galenic venous system [14]. Some other features of the CSF are summarized in Table 1.

**Table 1:** Characteristics of human, monkey, rat, and mice CSF in physiological conditions

	Human	Monkey	Rat	Mice
Total volume	150 ml [51]	10 ml [52]	Ranges from 90 to 300 μl [53, 54]	0.04 ml [44, 54]
Production rate	0.35 ml/min [10]	29–41 μl/min [54]	2.1–5.4 μl/min [44, 54]	0.325 μl/min [44, 54]

## Circulation of CSF

The steady CSF flow, caused by the CSF production mainly in the choroid plexus of the ventricles, is superimposed by a pulsatile motion, which is synchronized with the cardiac cycle.

### Major CSF Pathway

**Fig. 1:**
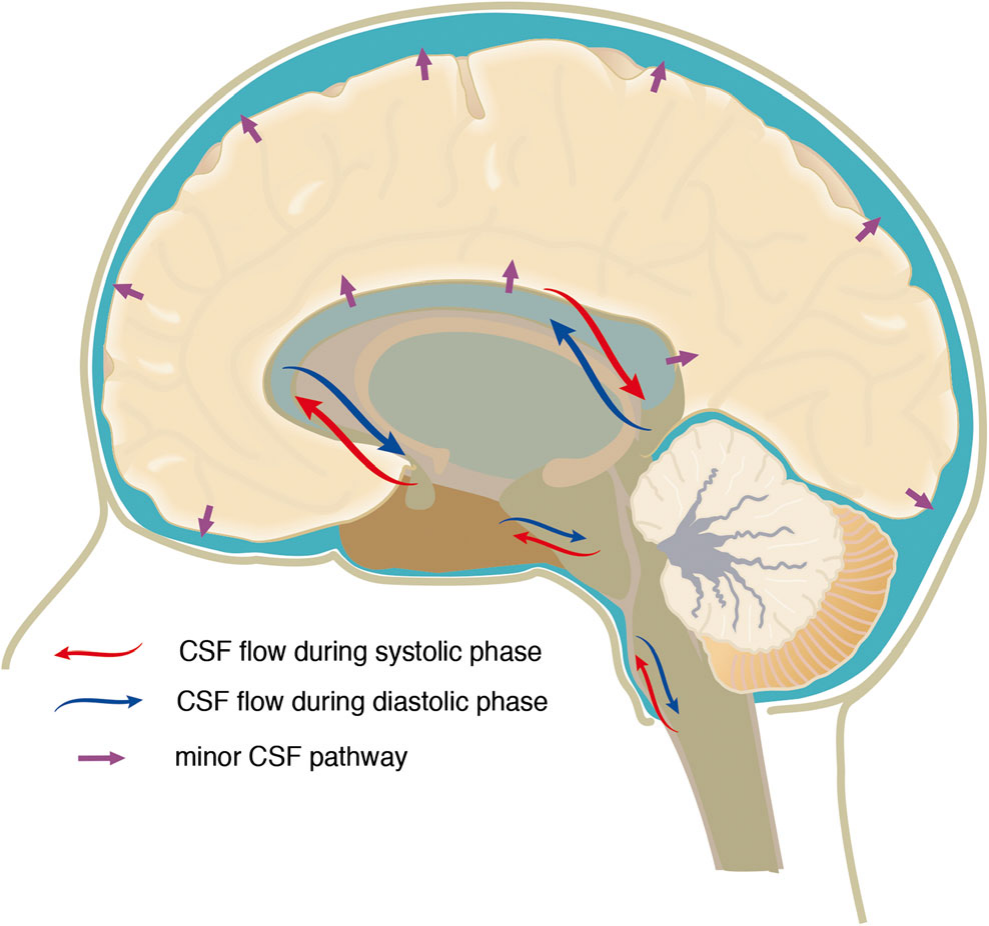
Schematic representation of the major CSF pathways. The major pathway follows the cardiac rhythm, while the minor pathway is described as a constant flow of CSF through the ependyma towards the subarachnoid space [15]

During the systolic phase, the CSF flows in cranio-caudal direction from the lateral ventricles to the third ventricle via the foramina of Monro and through the aqueduct of Sylvius into the fourth ventricle where it leaves the ventricle via the foramina of Magendie and Luschka into the subarachnoid space. Conversely, during diastole, CSF flows in the same route but in reverse direction [15] (Fig. 1). The unidirectional flow of the CSF is stimulated by arachnoid granulations, as shown by an in-vitro model of CSF outflow perfusion study in humans [16]. This undisturbed and unidirectional flow of the CSF is described as an important factor in maintaining homeostasis in CNS.

According to a time-resolved three-dimensional magnetic resonance velocity mapping study, the maximum peak value of the velocity vector for CSF flow in the lateral ventricles ranged between 0.3 and 1.3 cm/s (mean ± SD 0.7 ± 0.2 cm/s), while the mean value ranged between 0.1 and 0.3 cm/s (0.2 ± 0.1 cm/s) [15]. This study including 40 healthy volunteers determined that CSF flow in the ventricular system is a three-dimensional pattern just as blood flows in the vascular system. A consistent CSF flow pattern was found in the lateral ventricles: one part of CSF flows towards the foramina of Monro and moves caudally through the ventricular system, while the other part remains in the lateral ventricles and moves in rostral direction. The latter suggests that a substantial exchange of molecules across the ependymal layer might occur during the time that the CSF (and possibly the dissolved molecules in it) stays in the ventricle.

In the third ventricle, the CSF flows in a left-right direction when it comes from and goes into the foramina of Monro. This pattern was observed in the vortices of the posterior part of the third ventricle, but not in the central part. Inside the fourth ventricle, the CSF flow patterns tend to be more complex when compared with the lateral and third ventricles, mainly due to involvement of significant motion in all three dimensions and complexity of the fourth ventricle’s structure.

Of note, the abovementioned CSF flow patterns are characteristic for young and healthy subjects. It has been shown that the magnitudes of the frequency components of CSF flow in the aqueduct differ significantly between young and elderly subjects, as do the frequency components of the cervical spinal CSF and the arterial flows [17, 18]. In particular, total cerebral blood flow decreases with age, whereas the arteriovenous delay is preserved. Furthermore, CSF stroke volumes are significantly reduced in the elderly, at both aqueductal and cervical levels, as a result of arterial loss of pulsatility [18]. The most intuitive potential causes for the observed differences between the young and elderly groups are vascular changes and brain atrophy [17].

### Minor CSF Pathway

**Fig. 2:**
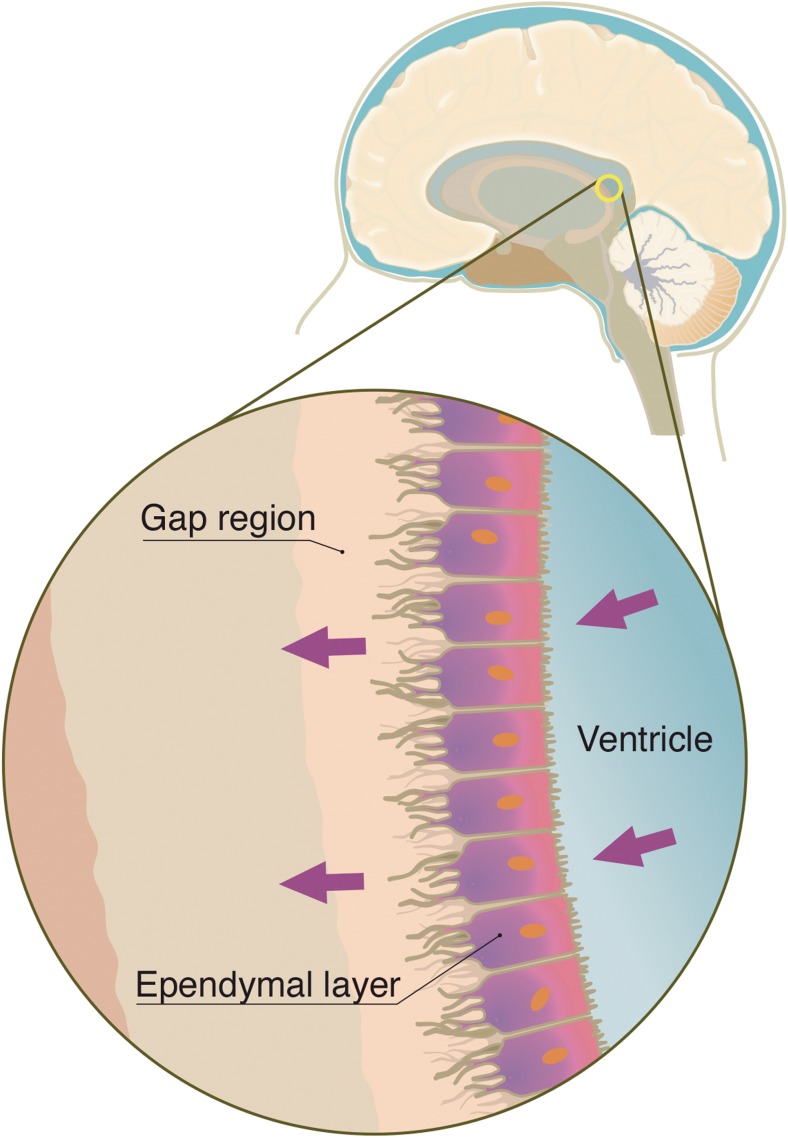
Schematic representation of the minor CSF pathway. Minor pathway is described as a constant flow of CSF through the ependyma towards the subarachnoid space [14]

Besides the major pathway, a minor pathway has been described for CSF flow. The minor pathway is a route that the CSF makes through the ventricular ependymal layer, interstitial and perivascular space, and perineural lymphatic channels [14] (Fig. 2). Unlike in humans, the minor CSF pathway seems to play a prominent role in other mammals. It is well known that mouse, rat, rabbit, and cat do not have arachnoid granulation (pacchionian bodies) through their entire lifespan [14]. In humans and large mammals, arachnoid granulation functions as the CSF reabsorption route in later age. For mammals without arachnoid granulation, the minor CSF pathway contributes to the maintenance of CSF dynamics.

Despite being described as minor pathway, this channel of CSF circulation can play a significant role in ICV drug delivery, which is the main topic of this article. The existence of a semipermeable barrier between the CSF and extra-cellular spaces has been known for some years and allows mixing of interstitial fluid and CSF [19]. This ependyma is formed by a single layer cuboidal to columnar ciliated epithelium, in which cells are connected by zonula adherens and have abundant gap junctions [19]. Due to the permeability observed at this layer, a continuous perivascular flow into the parenchyma mandates an outflow current of CSF [20], henceforth known as minor pathway.

## Transependymal CSF Flow

The use of CSF as delivery and carrier substrate for CNS drugs is a common practice in several pathologies. Eventually, the drugs infused intra-CSF are expected to reach their sites of action, most of the time, deep structures or tumors. However, how these infused compounds cross the ependymal layer and reach the parenchyma remains to be elucidated. Transependymal flow can simply be defined as a motion of fluids/molecules across the ependymal layer of cells that surrounds the ventricles and consequently penetration of this liquor into the brain parenchyma and adjacent structures.

Recently, the contribution of different structures in secreting and removing CSF was determined, and evidence of how fluid secreted into the brain may move through the brain parenchyma and thence exit to the periphery was collected [21]. Two main mechanisms are suggested to explain this process: diffusion and bulk flow. Diffusion is defined as an observable movement of substances due to their random thermal motions, while bulk flow is the movement of a fluid where all components of the fluid tend to move together. Diffusion is the main process over short distances, i.e., less than a few microns and sometimes up to few millimeters when a high-concentration gradient exists. Over long distances, bulk flow is the dominant process [21]. The concept of transependymal flow entails a combination of both mechanisms, since it accommodates distance as a factor.

### Early Indirect Evidence for Transependymal CSF Flow

Transependymal CSF flow can be explained partially by the studies done on experimental hydrocephalus models during the 60s. Early evidence is provided by animal studies: using radioactive-labeled substances, it turned out that the infused compound crosses the periventricular ependymal layer towards the extracellular space [22, 23]. Later, in a dog model of chronic communicating hydrocephalus, James and co-workers used a radioactive isotope of [^131^I] serum albumin, which was slowly infused intraventricularly, and animals were subjected to autoradiography. After injecting the radiolabeled compound into the cisterna magna of normal animals, radioactivity was observed in the basilar cisterns that spread over to the cerebral cortex [24]. At necropsy, the ventricles of the animals with communicating hydrocephalus were clearly enlarged, mainly at the anterior and occipital region of the lateral ventricles. These findings were thought to be related to the high intracranial pressure observed in communicating hydrocephalus, despite the fact that the high pressure per se does not explain why the radiolabeled substances where found in the cerebral cortex. By contrast, no enlargement was observed in control animals although autoradiographs revealed few sub-ependymal radioactivity and minimal periventricular radioactivity. Detailed analyses revealed that there is a facilitated diffusion—bulk flow—of the [^131^I] serum albumin across the ependymal layer of the ventricular system. Moreover, there was a reduced gradient of the isotope from the ependymal surface and the brain parenchyma of animals with chronic communicating hydrocephalus. In this condition, the ventricular ependymal layer suffers histological damages leading to changes in its permeability to large molecules. Based on these results, it was suggested that in experimental conditions, the ventricular walls might be an alternative route for CSF, although this idea had been brought up earlier as well [25]. Seemingly, this study proves the existence of an alternative passage for CSF in pathological conditions characterized by impaired CSF circulation. A “reverse” flow of CSF was suggested to be the reason behind the wide distribution of the compound. This reverse flow would allow the labeled albumin to cross the basal cisterns towards the lateral ventricle. However, this theory needs to be treated with caution, since the proposed reverse flow of CSF has not been backed up by scientific evidence, yet.

### Recent Evidences

Since CSF drainage is impaired in normal pressure hydrocephalus (NPH), an alternative way is the leakage of CSF into the brain parenchyma, described as periventricular lucency [26]. Lucency refers to hypodense areas, especially around the horns of the lateral ventricles. After injecting a radioactive tracer into the lumbar space, it is commonly seen that the tracer accumulates in the ventricles, providing indirect evidence that CSF is absorbed into the brain parenchyma [27].

Several speculations have been put forward to explain the mechanism behind lucency. The main theory suggests a disruption of the ependymal wall and extravasation of CSF [28]. Recent findings, using a finite element model of a hydrocephalic brain, are in line with clinical data showing that periventricular lucency is more prevalent in moderate ventriculomegaly than in severe ventriculomegaly [29]. However, this observation was not regarded as definitive counterevidence to the CSF extravasation theory, since they only showed that periventricular lucency can occur without rupture of the ependymal wall.

Some other processes have been invoked to explain these findings, like the passive process of diffusion. Simple diffusion through a permeable ependymal layer cannot be the mechanism behind the transependymal crossing of the large albumin molecules. For instance, histological and ultrastructural investigation on the brains with periventricular oedema have shown that distinct dynamic processes are involved in CSF crossing through brain parenchyma [21]. In a different study, inulin or sucrose, substances that are bound to the extracellular space of the brain parenchyma, diffuses from the ventricle into the neuropil [22, 30].

The process of CSF absorption from the ventricular system into the parenchyma was initially suggested by DeLand and colleagues [31] and later reinforced by James and co-workers [24, 32]. Proteins and large size molecules can be taken by CSF from the ventricular system into the brain parenchyma in normal and hydrocephalic animals [30, 31].

More recently, a study reported that low-dose acetazolamide, frequently used to treat cerebral oedema and increased intracranial pressure, was able to reduce periventricular lucency in idiopathic NPH [33]. The reduction of the lucency happened almost entirely within the periventricular region, which is consistent with a decrease in transependymal CSF flow due to the administration of acetazolamide. Possibly, a diuretic medication reduces cerebral oedema through a declined transependymal flow.

With the advent of more advanced techniques, like magnetic resonance imaging (MRI), the dynamics of CSF could be studied in more detail and some new facts were brought to light. Clinical application of MRI demonstrated that under high intraventricular pressure, “water” penetrates into the periventricular tissue [34, 35]. In this study, patients with ventricular obstruction, or with idiopathic intracranial hypertension, were subjected to MR scanning. MRI signal revealed transependymal passage of CSF, and possibly due to high intracranial pressure, CSF is pulled across the ependymal layer into the extracellular space of the surrounding white matter. This transependymal passage of CSF flow, occurring under abnormal conditions such as idiopathic intracranial hypertension, was regarded as an unusual process of CSF outflow [9].

To investigate regional distribution and kinetics in the brain, a physiologically based pharmacokinetic modelling approach was applied in rats, using acetaminophen as a test compound [36]. This compound is not subjected to active transport processes, allowing quantification of regional diffusion and fluid flow processes within the brain. Concentration-time profiles of the brain extracellular fluid, CSF in lateral ventricles, and CSF in cisterna magna showed a pattern similar to the unbound plasma concentration-time profile, pointing to a relatively quick distribution of the compound. The compound concentration in CSF was found to be similar to both the lateral ventricles and cisterna magna; however, the concentration in the brain extracellular fluid was on average fourfold higher than CSF in the same locations [36]. It was thought that due to a lack of physical barrier between the CSF and brain extracellular fluid, any concentration gradient balances readily [36–39]. This in fact ignores the existence of the ependyma as a physical barrier. Besides, it seems to be in contrast to what has been reported previously, since the concentration of the compound in the brain extracellular fluid was found to be fourfold higher than the concentration in CSF. This difference was explained by the relatively high turnover rate of CSF rather than the existence of a barrier between the two fluids [36]. By assuming the existence of a physical barrier—the ependyma—the difference in concentrations between the extracellular fluid and CSF can be explained by transependymal flow of the CSF. Meaning that, this flow constantly transports the infused compound from the ventricles and accumulates it in the parenchyma. This process is not in contrast with none of the previous theories stating that the concentrations across CSF and brain extracellular fluid balance readily and the high turnover of CSF maintains a low-concentration gradient in the ventricles.

### CSF Flow Revisited

In this section, we will “revisit” previous studies and apply our current knowledge on transependymal CSF flow to elucidate their findings. As mentioned before, two main mechanisms are suggested to explain how fluid secreted into the brain may move through the brain parenchyma and thence exit to the periphery: diffusion and bulk flow. Although, diffusion can explain a part of the process of how substances can cross the ependymal layer, the movement of compounds across long distances still needs to be clarified. As an intermediate process, transependymal CSF flow has been hypothesized to be the main process that drives substances inside the brain, instead of just diffusion or bulk flow [21]. This intermediate process would explain why those compounds can cross the ependymal layer and be found in remote structures and regions of the brain parenchyma. Of note, various studies use distinct terminology to illustrate the possible process of transependymal flow. Because terms like diffusion, transport, facilitated diffusion, and migration are used interchangeably across literature, we kept the original terms as described in the primary sources and use the term transependymal CSF flow consequently.

The ventricular ependymal layer contains fewer tight gap junctions, allowing the CSF and extra-cellular fluid to communicate and even albumin and other proteins to cross this layer [13]. Albumin is a protein that originates from blood and is able to enter CSF in the ventricles, cisterns, or lumbar and cortical subarachnoid space [13]. One of the first described experimental studies available looking at the permeability of the ependymal lining states clearly that the ependyma is not a tight barrier [40]. In this study, insulin was used to measure the permeability of the ependymal layer, showing that insulin could cross the ependyma and was found in the caudate nucleus and midbrain. At the same time, there was no increased concentration of a weak organic acid - para-aminohippurate (PAH)- which is known to be subject of active transport in the sub-ependymal layer, suggesting that the transport across the ependymal layer is not of an active kind.

Moreover, CSF is mainly produced in the lateral ventricles [41], which creates a driving flow throughout all the ventricular system. Recently, imaging studies have provided valuable information about the fluid dynamics inside the ventricular system. According to Stadlbauer and colleagues [15], a part of the CSF stays in the lateral ventricles and moves anteriorly, while the remaining CSF flows to the third ventricle and so on. Since a part of CSF remains in the lateral ventricles, “bathing” the ependymal layer and increasing the time for exchange of fluids between the ventricle and brain parenchyma predicts a transependymal CSF flow.

Since the CSF pressure gradient is higher than the pressure in the venous sinuses [11, 12], CSF can cross the ependymal layer of cells, which circumvent the lateral ventricles, and flow in direction of the venous sinuses, into which it is eventually absorbed. Therefore, it is not unexpected that on the way from the ventricles to the venous sinuses, CSF crosses all the parenchyma and adjacent structures, carrying the dissolved molecules towards the parenchyma.

This pressure gradient might empirically explain why infusion in the subarachnoid space is not as effective, since this compound is administrated against the stream. This eccentric pressure also supports and explains why ICV is a preferred approach over other ways of drug delivery. This higher CSF pressure, in comparison with the venous blood, might be a factor in supporting the CSF transport process proposed here, transependymal CSF flow.

The high intraventricular pressure explains the accumulation or absorbance of substances around the horns of the lateral ventricles in hydrocephalus. However, based on MRI studies, high intracranial pressure cannot explain per se why CSF periventricular leakage is observed in patients with chronic obstructive hydrocephalus [42]. This piece of evidence points once again towards transependymal CSF flow.

## Direct Drug Delivery

The physiological constitution and flow of CSF makes this fluid a suitable vehicle for drugs/compounds across the whole brain. Although transependymal flow has not been clearly defined, several clinical and research methods of ICV drug delivery such as antisense oligonucleotide (AON) or oligodeoxynucleotide (ODN) therapies have recently provided indirect evidences supporting the existence of a transependymal CSF flow.

For instance, RNA interference can silence mutant *huntingtin* throughout most brain regions and ameliorate Huntington’s disease symptoms in rodents and nonhuman primates by exploiting the natural flow of CSF to deliver AONs widely after ICV infusion. As a matter of fact, ICV infusion of the AON appears to be the most effective method and achieves a broad distribution in the nonhuman primate brain [5].

**Fig. 3:**
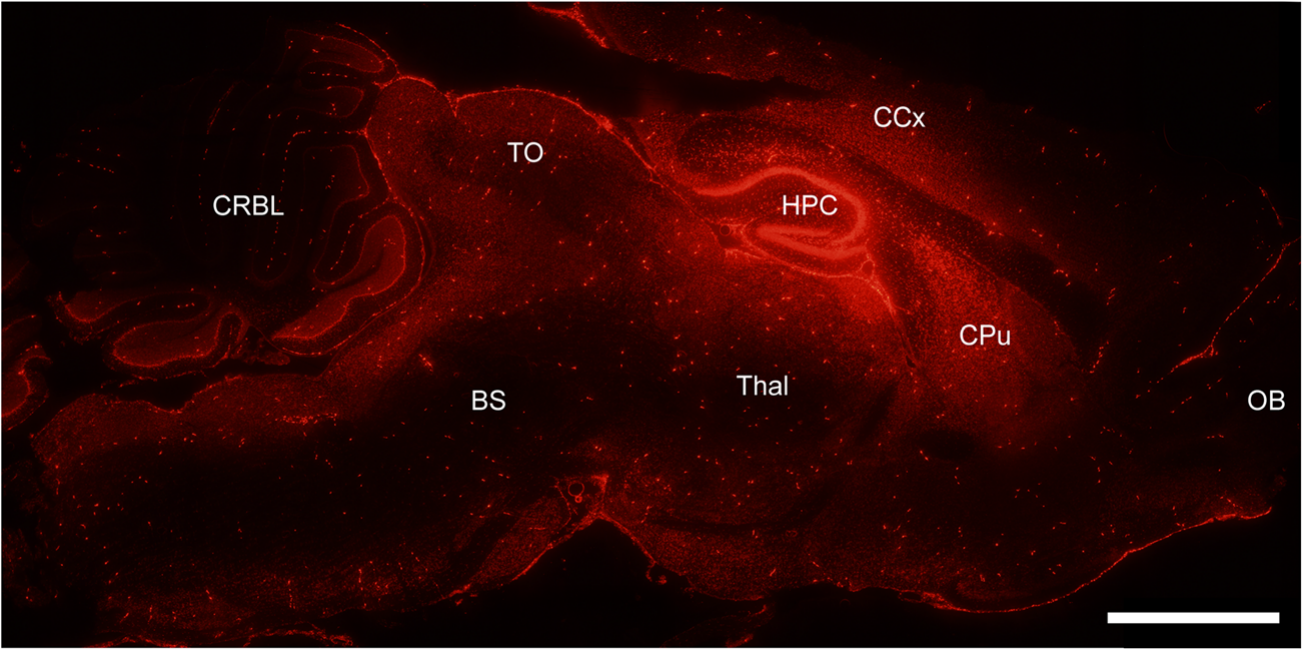
Low-power photomicrograph taken from a sagittal section of a mouse brain infused with fluorescent-labeled oligonucleotide and perfused after 24 h. Image shows distribution of the oligonucleotide throughout the brain after a single infusion. Neocortex (*CCx*), tectum opticum (*TO*), thalamus (*Thal*), caudate-putamen (*CPu*), hippocampus (*HPC*), olfactory bulb (*OB*), brain stem (*BS*), and cerebellum (*CBRL*). *Scale bar* 1500 μm

Our data shows that a single infusion of a fluorescent-labeled oligonucleotide into the lateral ventricle of naive mice is followed by its distribution in many brain regions after 24 h including the cerebellum, brain stem, striatum, cortex, and hippocampus [43] (Fig. 3).

In an experiment by Grzanna and co-workers, fluorescein-labeled ODN was delivered into the lateral ventricle in rats. Twenty-four hours after the infusion, the tissue adjacent to the lateral ventricle on the contralateral side was labeled, although less markedly than the ipsilateral side of the injection. In line with previous observations, the penetration of ODNs in the tissues around the third and fourth ventricles was less than around the lateral ventricles due to increased distance from the injection site [4]. Besides periventricular tissues, areas facing the subarachnoid space were also found to be labeled with biotinylated ODNs, such as the outer layers of the cerebral cortex, superficial layer of the superior colliculus, lateral geniculate, and outer regions of the cerebellum [4]. These results sustain the concept in which compounds can cross the ependymal layer around the ventricle, as well as spread throughout the brain tissue, possibly using the extra-cellular fluid as a surrogate. Although, some have stated that drugs injected into the CSF compartment do not distribute to the inner parenchyma of the brain [44]. It is plausible that this discrepancy could be due to the use of specific substances or pathological condition of subjects that underwent the procedure.

Diffusion might be the process behind the distribution of the labeled substances, and in general of the therapeutic agents, yet this would only be valid for short distances. What explains distribution of the substances in the outer layers of the cortex, cerebellum, etc.? After crossing the ependymal layer, the substances are carried throughout the parenchyma in a process similar to bulk flow, following the drainage route of the CSF in direction to the venous sinuses.

All together, these studies point towards a common process, in which intraventricular delivery of the compound can eventually affect the target site, for instance striatum, hippocampus, etc. Seemingly, transependymal CSF flow is the most probable process to explain these results. The ependymal layer that surrounds the ventricles is permeable to AON and allows AON to cross and spread across the parenchyma. Moreover, it has less tight junctions than a normal ependymal layer would have [19]. Such a “leaky” barrier would not be enough to explain the distribution of the ICV-infused compounds across the parenchyma of the brain. The CSF follows certain flow patterns, which happen to be in an outward direction, i.e., from the ventricle to the superior sinuses. This natural flow allows the molecules that crossed the ependymal layer to reach the structures within the parenchyma. Moreover, this directional flow leads to even distribution of the infused drug/compound, while intrathecally delivered agents often face slow, incomplete, and uneven distribution in the brain [45].

Despite the positive results mentioned above, some limitations to the translational feature of this study were identified. These include a need to incorporate a device that allows repeated and chronic infusions of AONs into the CNS and also the necessity to distribute the compound properly in order to target regions in a larger brain [7]. These pitfalls can be easily overcome by using devices such as the Ommaya reservoir [6]. Moreover, since it was reported that AONs were found in regions of the nonhuman primate brain such as the caudate nucleus, cortex, and cerebellum, these obstacles can be easily overcome.

A compound delivered locally into CSF can be affected by the distance from the CSF to the site of action, even if there are only a few centimeters. If the drug is expected to diffuse/spread and penetrate the brain tissue, the infused dose needs to be sufficient to reach the site of action. Brain regions in contact with CSF and potentially other regions are best reached by repeating the infusions or continuous delivery of compounds into the lateral ventricle [4]. Besides, the microvessels produce interstitial fluid at a low but finite rate, which creates a flow towards CSF spaces, e.g., ventricles, in opposite direction of diffusive drug penetration. Lastly, the high turnover rate of CSF (in humans, total volume renews every 5–6 h) is transduced in a rapid cleaning of the infused drug back into the blood circulation. These criteria dictate that infusion of drugs might only be suitable to targets close to the ventricles [45].

## Clinical Significance

Direct drug delivery into the CNS is achievable via three main approaches that are developed in order to circumvent the existence of the BBB: intrathecal, intraparenchymal, and intraventricular delivery. The last two approaches are frequently preferred over intrathecal injection, since this method does usually not allow deep penetration of the substance in the brain which is frequently ineffective and can cause serious side effects. Moreover, substances delivered intrathecally are distributed unevenly, slowly, and incompletely in the brain [45]. As an alternative, drug delivery into the ventricles or brain parenchyma is a clinically relevant approach for chemotherapy. A synthetic device (Ommaya reservoir) is placed subcutaneously on the scalp, from which an outlet catheter delivers the drug into the ventricles—intraventricular infusion—within the brain [45].

In 1963, the Ommaya reservoir was described as a subcutaneous reservoir and pump for sterile access to ventricular cerebrospinal fluid. Even with empirical data, the use of this approach is recommended by stating that “distribution of substances in the CSF depends largely on the site of introduction: the substance mixes best when introduced into the lateral ventricles” [6].

Another technique widely used in treating tumors that may provide some understanding on how drugs can be delivered locally is convection-enhanced delivery (CED). CED was developed by Edward Oldfield and colleagues in the early 1990s, as a method to distribute large and small molecular weight compounds across the BBB (reviewed by [46]). This method allows robust distribution of the infused compound at the infusion site and is an effective treatment for brain tumors and other pathologies of the CNS [46]. On the basis of this approach, bulk flow is driven by a small hydrostatic pressure derived by maintaining the pressure gradient at the catheter tip using a syringe pump [46]. Notably, due to recent advances in stereotactic and functional neurosurgery, surgical procedures and targeting issues have been overcome and these types of surgeries can be conducted in any average neurosurgical setting.

It should be noted that similar to every surgical procedure, both techniques face some shortcomings in clinical application. Ommaya reservoir can lead to a slow rate of drug distribution within the CSF, enhanced intracranial pressure, or CSF leakage [47]. When using CED, difficulties in achieving consistently accurate delivery to the desired volume or leakage of the infusate into the ventricles or sulci and/or reflux along the catheter tract could occur [48].

### Future Perspectives

Current methodological and scientific advances in ICV drug delivery together with abovementioned scientific data predicts an upsurge in implementation of this approach in clinical practice. The transependymal CSF flow seems to be the basis of this approach, even if not clearly recognized as a physiological process thus far. Directional flow of the CSF from the lateral ventricles—the main production site—might allow the infused therapeutics to be carried and distributed to distant brain regions from the site of injection.

Taking into account, this process will pave the road to benefit from ICV drug delivery advantages, as such crossing the blood-brain and blood-CSF barriers with a minimal risk of systemic effects and uniform distribution of the compound in the parenchyma and subarachnoid space [49, 50].

The development of local drug delivery into the CNS was born as response to circumvent the protective features of the BBB. What once was considered to be an obstacle turns out to be an advantage in ICV drug delivery. The BBB prevents the therapeutic from leaking out, reducing the possible risk of systemic effects by keeping the drug in its site of action.

### Conclusions

The aim of the present article was to provide a synopsis on the available evidence for a process of transependymal CSF flow. This form of CSF flow exists and plays a significant role in CSF physiology. It provides an attractive route for drug delivery. We believe that this approach will open new opportunities for investigating drug-based therapeutics for neurological and psychiatric disorders.

## References

[1] Global Burden of Disease Study C (2015). Global, regional, and national incidence, prevalence, and years lived with disability for 301 acute and chronic diseases and injuries in 188 countries, 1990-2013: a systematic analysis for the Global burden of disease study 2013. Lancet.

[2] Begley DJ (2004). Delivery of therapeutic agents to the central nervous system: the problems and the possibilities. Pharmacol Ther.

[3] Lu CT, Zhao YZ, Wong HL, Cai J, Peng L, Tian XQ (2014). Current approaches to enhance CNS delivery of drugs across the brain barriers. Int J Nanomedicine.

[4] Grzanna R, Dubin JR, Dent GW, Ji Z, Zhang W, Ho SP, Hartig PR (1998). Intrastriatal and intraventricular injections of oligodeoxynucleotides in the rat brain: tissue penetration, intracellular distribution and c-fos antisense effects. Brain Res Mol Brain Res.

[5] Kordasiewicz HB, Stanek LM, Wancewicz EV, Mazur C, McAlonis MM, Pytel KA, Artates JW, Weiss A (2012). Sustained therapeutic reversal of Huntington’s disease by transient repression of huntingtin synthesis. Neuron.

[6] Ommaya AK (1963). Subcutaneous reservoir and pump for sterile access to ventricular cerebrospinal fluid. Lancet.

[7] Stanek LM, Sardi SP, Mastis BM, Richards AR, Treleaven CM, Taksir TV, Misra K, Cheng SH (2014). Silencing mutant huntingtin by AAV-mediated RNAi ameliorates disease manifestations in the YAC128 mouse model of Huntington’s disease. Hum Gene Ther.

[8] Symss NP, Oi S (2013). Theories of cerebrospinal fluid dynamics and hydrocephalus: historical trend. J Neurosurg Pediatr.

[9] Kapoor KG, Katz SE, Grzybowski DM, Lubow M (2008). Cerebrospinal fluid outflow: an evolving perspective. Brain Res Bull.

[10] Czosnyka M, Czosnyka Z, Momjian S, Pickard JD (2004). Cerebrospinal fluid dynamics. Physiol Meas.

[11] Friden HG, Ekstedt J (1983). Volume/pressure relationship of the cerebrospinal space in humans. Neurosurgery.

[12] Blomquist HK, Sundin S, Ekstedt J (1986). Cerebrospinal fluid hydrodynamic studies in children. J Neurol Neurosurg Psychiatry.

[13] Reiber H (2003). Proteins in cerebrospinal fluid and blood: barriers, CSF flow rate and source-related dynamics. Restor Neurol Neurosci.

[14] Oi S, Di Rocco C (2006). Proposal of “evolution theory in cerebrospinal fluid dynamics” and minor pathway hydrocephalus in developing immature brain. Child’s nervous system: ChNS: official journal of the International Society for Pediatric Neurosurgery.

[15] Stadlbauer A, Salomonowitz E, van der Riet W, Buchfelder M, Ganslandt O (2010). Insight into the patterns of cerebrospinal fluid flow in the human ventricular system using MR velocity mapping. NeuroImage.

[16] Grzybowski DM, Holman DW, Katz SE, Lubow M (2006). In vitro model of cerebrospinal fluid outflow through human arachnoid granulations. Invest Ophthalmol Vis Sci.

[17] Schmid Daners M, Knobloch V, Soellinger M, Boesiger P, Seifert B, Guzzella L, Kurtcuoglu V (2012). Age-specific characteristics and coupling of cerebral arterial inflow and cerebrospinal fluid dynamics. PLoS One.

[18] Stoquart-ElSankari S, Balédent O, Gondry-Jouet C, Makki M, Godefroy O, Meyer M-E (2007). Aging effects on cerebral blood and cerebrospinal fluid flows. J Cereb Blood Flow Metab.

[19] Del Bigio MR (1995). The ependyma: a protective barrier between brain and cerebrospinal fluid. Glia.

[20] Brodbelt A, Stoodley M (2007). CSF pathways: a review. Br J Neurosurg.

[21] Hladky SB, Barrand MA (2014). Mechanisms of fluid movement into, through and out of the brain: evaluation of the evidence. Fluids and barriers of the CNS.

[22] Sahar A, Hochwald GM, Sadik AR, Ransohoff J (1969). Cerebrospinal fluid absorption in animals with experimental obstructive hydrocephalus. Arch Neurol.

[23] Bowsher D (1957). Pathways of absorption of protein from the cerebrospinal fluid: an autoradiographic study in the cat. Anat Rec.

[24] James AE, Strecker EP, Sperber E, Flor WJ, Merz T, Burns B (1974). An alternative pathway of cerebrospinal fluid absorption in communicating hydrocephalus. Transependymal movement. Radiology.

[25] Brightman MW (1968). The intracerebral movement of proteins injected into blood and cerebrospinal fluid of mice. Prog Brain Res.

[26] Gjerris F, Snorrason E (1992). The history of hydrocephalus. J Hist Neurosci.

[27] Larsson A, Moonen M, Bergh AC, Lindberg S, Wikkelso C (1990). Predictive value of quantitative cisternography in normal pressure hydrocephalus. Acta Neurol Scand.

[28] Serdar M (1995). Periventricular lucency on computed tomography associated with hydrocephalus: what is the cause?. Surg Neurol.

[29] Kim H, Jeong EJ, Park DH, Czosnyka Z, Yoon BC, Kim K, Czosnyka M, Kim DJ (2015) Finite element analysis of periventricular lucency in hydrocephalus: extravasation or transependymal CSF absorption? J Neurosurg:1–8. doi:10.3171/2014.11.jns141382 10.3171/2014.11.JNS141382 26274984

[30] Strecker EP, James AE, Kelley JE, Merz T (1974). Semiquantitative studies of transependymal albumin movement in communicating hydrocephalus. Radiology.

[31] DeLand FH, James AE, Ladd DJ, Konigsmark BW (1972). Normal pressure hydrocephalus: a histologic study. Am J Clin Pathol.

[32] James AE, Strecker EP (1973). Use of silastic to produce communicating hydrocephalus. Investig Radiol.

[33] Alperin N, Oliu CJ, Bagci AM, Lee SH, Kovanlikaya I, Adams D, Katzen H, Ivkovic M (2014). Low-dose acetazolamide reverses periventricular white matter hyperintensities in iNPH. Neurology.

[34] Gideon P, Thomsen C, Gjerris F, Sorensen PS, Henriksen O (1994). Increased self-diffusion of brain water in hydrocephalus measured by MR imaging. Acta radiologica (Stockholm, Sweden: 1987).

[35] Sorensen PS, Thomsen C, Gjerris F, Henriksen O (1990). Brain water accumulation in pseudotumour cerebri demonstrated by MR-imaging of brain water self-diffusion. Acta Neurochir Suppl.

[36] Westerhout J, Ploeger B, Smeets J, Danhof M, de Lange EC (2012). Physiologically based pharmacokinetic modeling to investigate regional brain distribution kinetics in rats. AAPS J.

[37] de Lange EC, Danhof M (2002). Considerations in the use of cerebrospinal fluid pharmacokinetics to predict brain target concentrations in the clinical setting: implications of the barriers between blood and brain. Clin Pharmacokinet.

[38] Shen DD, Artru AA, Adkison KK (2004). Principles and applicability of CSF sampling for the assessment of CNS drug delivery and pharmacodynamics. Adv Drug Deliv Rev.

[39] Lin JH (2008). CSF as a surrogate for assessing CNS exposure: an industrial perspective. Curr Drug Metab.

[40] Rall DP (1968). Transport through the ependymal linings. Prog Brain Res.

[41] de Lange EC (2013). Utility of CSF in translational neuroscience. J Pharmacokinet Pharmacodyn.

[42] Manara R, Citton V, Traverso A, Zanotti MC, Faggin R, Sartori S, Perini R, Milanese L (2015). Intraparenchymal ventricular diverticula in chronic obstructive hydrocephalus: prevalence, imaging features and evolution. Acta Neurochir.

[43] Casaca-Carreira J, Temel Y, Larrakoetxea I, Jahanshahi A (2016). Distribution and penetration of intracerebroventricularly administered 2'OMePS oligonucleotide in the mouse brain. Nucleic acid therapeutics.

[44] Pardridge WM (1991) Transnasal and intraventricular delivery. In: Peptide Drug Delivery to the Brain. Raven Press, p 112

[45] Pathan SA, Iqbal Z, Zaidi SM, Talegaonkar S, Vohra D, Jain GK, Azeem A, Jain N (2009). CNS drug delivery systems: novel approaches. Recent patents on drug delivery & formulation.

[46] Saito R, Tominaga T (2012). Convection-enhanced delivery: from mechanisms to clinical drug delivery for diseases of the central nervous system. Neurol Med Chir.

[47] Scheld WM (1989). Drug delivery to the central nervous system: general principles and relevance to therapy for infections of the central nervous system. Rev Infect Dis.

[48] Debinski W, Tatter SB (2009). Convection-enhanced delivery for the treatment of brain tumors. Expert Rev Neurother.

[49] Lombardi G, Zustovich F, Farina P, Della Puppa A, Manara R, Cecchin D, Brunello A, Cappetta A (2011). Neoplastic meningitis from solid tumors: new diagnostic and therapeutic approaches. Oncologist.

[50] Gabay MP, Thakkar JP, Stachnik JM, Woelich SK, Villano JL (2012). Intra-CSF administration of chemotherapy medications. Cancer Chemother Pharmacol.

[51] Johanson CE, Duncan JA, Klinge PM, Brinker T, Stopa EG, Silverberg GD (2008). Multiplicity of cerebrospinal fluid functions: new challenges in health and disease. Cerebrospinal Fluid Res.

[52] Riva L, Blaney SM, Dauser R, Nuchtern JG, Durfee J, McGuffey L, Berg SL (2000). Pharmacokinetics and cerebrospinal fluid penetration of CI-994 (N-acetyldinaline) in the nonhuman primate. Clinical cancer research: an official journal of the American Association for Cancer Research.

[53] Davson H (1969) The cerebrospinal fluid. In: Handbook of Neurochemistry, vol 2. pp 23–48

[54] Yaksh TL (1999) Chapter 8, spinal cerebrospinal fluid chemistry and physiology. In: Artru AA (ed) Spinal drug and delivery. Elsevier sciences B.V., pp 177–238

